# Supplementary material for: Kallikrein-8 mediates furin-independent Activin-A precursor processing to stimulate tumor growth in melanoma
Source: Nat Commun. 2025 Mar 10;16:2354. doi: 10.1038/s41467-025-57661-5 (PMC11893775; doi:10.1038/s41467-025-57661-5)
Supplement: Supplementary file 1 — Supplementary Information [file 41467_2025_57661_MOESM1_ESM.pdf]

Supplemental Table 1. Primers for mutagenesis and Gateway cloning.

Primer	Sequence
del310 For	CGCCGGCGTCGGGGCTTGGAGCAGAAGCTG
del310 Rev	CTGCTCCAAGCCCCGACGCCGGCGATGAG
R310A For	CGCCGGCGTCGGGCCGGCTTGGAGCAGAAGCTG
R310A Rev	GGCCCGACGCCGGCGATGAGGGTGGTCTTCAGAC
RALE For	CGGGCCCTCGAGGAGCAGAAGCTGATATCCGAG
RALE Rev	CTTCTGCTCCTCGAGGGCCCGACGCCGGCG
α1-PDX For	GGGGACAAGTTTGTACAAAAAAGCAGGCTGCC GCCACCATGCCCTCGAGCGTCTC
α1-PDX Rev	GGGGACCACTTTGTACAAGAAAGCTGGGTCTA TTTTTGGGTGGGATTCACCAC

Supplemental Table 2. IPTG-inducible shRNA sequences.

**shRNA**	**Sequence**
shKlk8_1	CCCTCAAGAGAACTTTCCAAA
shKlk8_2	CCAGCATCCTTGCTACAACAA
shKlk8_3	CCAGAAGATCACAGTCACGAT
shKlk8_4	CAGTCACGATATAATGCTCAT
shLuciferase	CGCTGAGTACTTCGAAATGTC

Supplemental Table 3. RT-(q)PCR primers used to quantify cDNA of the genes.

Gene		Primer sequence	Species
mycINHBA	fw	CCGAGGAGGACCTGTGTGA	Homo sapiens
	rev	ATCCAGTCATTCCAGCCGATG	
INHBA	fw	AATCTCGAAGTGCAGCGT	Homo sapiens
	rev	GGAGAACGGGTATGTGGA	
KLK8	fw	AAGTGCACCGTCTCAGGC	Homo sapiens
	rev	TCCTCACACTTCTTCTGGGG	
Klk8	fw	GGGTGATCATAGCCTCCAGA	Mus musculus
	rev	TTCACTTCCGCACAGTTGAG	
Klk5	fw	TTCAGGCACTGGAGGACTTTCG	Mus musculus
	rev	CTCAGGGAACAGGGGCAAA	
Pcsk1	fw	TGATGATCGTGTGACGTGGG	Mus musculus
	rev	CACTCCAAGCCATCATCCAGT	
Pcsk2	fw	ACAGCCCCACTTTTCACTCC	Mus musculus
	rev	CAAAGGGGAGCTTTCGGACT	
Pcsk3	fw	TCCCCAGGATCTGGCCCTTA	Mus musculus
	rev	CGACCACCCATAGCAACCAG	
Pcsk4	fw	ACCCTGGGCCTGGAGAATAA	Mus musculus
	rev	GAGGGGACTGTGACTTTCCTG	
Pcsk5	fw	CCCGTAACAAGGGTCTTGGA	Mus musculus
	rev	TCCCTTGGCAGGATAATGGC	
Pcsk6	fw	CGGAAGATCGTCACCACAGA	Mus musculus
	rev	TTTATGCCCAGCTCCGTTGA	
Pcsk7	fw	CGAGAGTTTCCGTAGGGTGG	Mus musculus
	rev	CATCAGAACAGCAGGCTGGG	
Pcsk9	fw	GCGAATTATCCCAGCATGGC	Mus musculus
	rev	GTCACACTTGCTCGCCTGTC	
GAPDH	fw	ACTGAGGACCAGGTTGTCTCC	Mus musculus
	rev	GTTGGGATAGGGCCTCTCTTGC	
GAPDH	fw	GGAAATGAATGGGCAGCCGT	Homo sapiens
	rev	GGATCTCGCTCCTGGAAGATGG	

Supplemental Table 4. siRNAs targeting the candidate PCLPs emerging from the RNAi library screen.

**siRNA**	**Sequence**
Adamts10_1	GCAGAGAGCGCUAUGUGGA
Adamts10_2	GCACUCACUUCCUGCUGAA
Adamts10_3	ACAAGAUGAUGGUGGCCUA
Adamts10_4	CGUCCAUGGUGGUUACGUA
C1rl_1	GAGCUAAACUGGGCAACUU
C1rl_2	GACCAGACAGGGACACUUA
C1rl_3	GUGCUCAGCUACAUGGAUU
C1rl_4	GAGGGUGAUUGAAGGUAAA
CtsZ_1	GGAGAAAUGUGAACGGUGU
CtsZ_2	GACCUGCACUGAAUUCAAA
CtsZ_3	GAUAAUGGCAACAGAGAUG
CtsZ_4	GUACUGGAUUGUCCGAAAU
Furin_1	GAAAGUGAGCCAUUCGUAU
Furin_2	CGACCUAGCAGGCAAUUAU
Furin_3	GGACAUCGGCAAACGGCUA
Furin_4	CGAAUGGGUCCUAGAGAUU
Klk6_1	AAACACAACCUACGGCAAA
Klk6_2	GUGCUUGGUUCUUGCUAAA
Klk6_3	ACCGAUGUCUGCACUCAUA
Klk6_4	CCAGUCAAAUUCUCUAAAA
Klk8_1	CGAGAAACCUGGAGUCUAC
Klk8_2	GAUCACAGUCACGAUAUAA
Klk8_3	GCGAGAGACUGAUCUGUGG
Klk8_4	GAAGGUCGAGAGUGUAUAC
Plg_1	GGAGGUGUCUCGGACUGUU
Plg_2	GGGCAGAGCUAUCGGGGUA
Plg_3	GAGAUUCCAUCCUGCGAGU
Plg_4	GCACGAAGAAUAUAUCCGU
ntControl_1	UAGCGACUAAACACAUCAA
ntControl_2	UAAGGCUAUGAAGAGAUAC
ntControl_3	AUGUAUUGGCCUGUAUUAG
ntControl_4	AUGAACGUGAAUUGCUCAA

Supplemental Table 5. Sequences of custom short interfering RNAs.

siRNA	Sequence
Pcsk5	CUACUGUGGACUAAAGUACAUCAAA
Pcsk6	CUACUGUGGACUAAAGUACAUCAAA

Supplemental Table 6. Antibodies used for flow cytometry.

Reagent	Source	Clone	Reference	Dilution
CD11b BV711	BioLegend	M1/70	101241	1:200
CD3 PE	BioLegend	145-2C11	100308	1:200
CD4 BV785	BioLegend	RM4-5	100552	1:200
CD45.2 BUV737	BD Horizon	104	564880	1:200
CD8 BV510	BD Horizon	53-6.7	563068	1:200

Supplemental figure legends

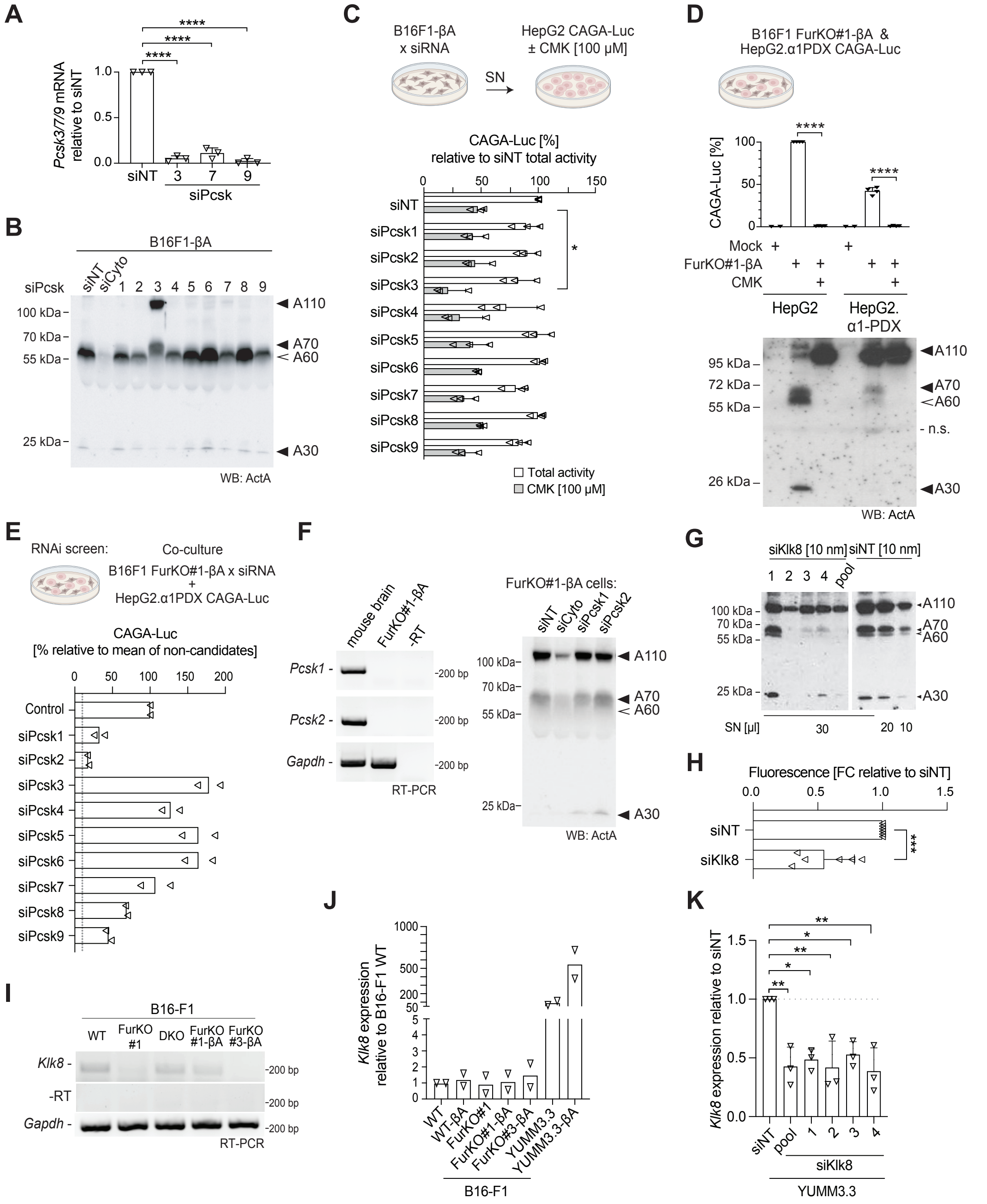

Figure S1. Influence of proprotein convertases and their inhibitors on Activin-A precursor processing and signaling.

A) RT-qPCR of *Pcsk* relative to *Gapdh* mRNA levels in B16F1-βA cells 3 d after siRNA transfection. Data represent means ±SD of three experiments (Two-sided Student's t-tests). **B)** Activin-A Western blot of SNs from siRNA-transfected B16F1-βA cells conditioned during 3 d. **C)** CAGA-Luc induction in HepG2 reporter cells 12 hrs after adding SNs from (B) ±100 µM CMK. Values were normalized to total signaling activity of siNT without CMK. Data represent means ±SD of three experiments (One-way ANOVA). **D)** CAGA-Luc induction in HepG2.α1PDX co-cultures with B16F1 FurKO#1-βA cells ±100 µM CMK. Values were normalized to the signaling activity in co-cultures of B16F1 FurKO#1-βA with control

HepG2 cells. Below: Activin-A in the SN after co-culturing for 4 d. Data represent means ±SD of four experiments (One-Way ANOVA). **E)** CAGA-Luc induction in HepG2.α1-PDX cells within 24 hrs after co-culturing with B16F1 FurKO#1-βA cells transfected with *Pcsk* siRNAs. Values represent means from two biological replicates after knockdown relative to the average CAGA-Luc induction by all other SNs ("non-candidates"). **F)** Left: Representative RT-PCR of *Pcsk1/2* mRNAs in B16F1 FurKO#1-βA and mouse brain (n=2). Right: Activin-A in SNs from B16F1 FurKO#1-βA cells conditioned during 3 d after transfection with siPcsk1 or siPcsk2. **G)** Western blot of Activin-A secreted by B16F1 FurKO#1-βA cells 3 d after transfection with the indicated siRNAs as in figure 1H, but after loading up to 30 µl of SNs. **H)** Alamar Blue staining of B16F1 FurKO#1-βA cells after siNT or siKlk8 transfection. Data show the average of six experiments ±SD (Two-sided Student's t-test). **I)** RT-PCR of *Klk8* in B16-F1 cell lines. Data is representative of three independent experiments with similar results. **J)** RT-qPCR of *Klk8* relative to *Gapdh* in the YUMM3.3 melanoma cells. Values represent means of two experiments, normalized to the expression in *Furin* WT B16-F1 cells. **K)** RT-qPCR of *Klk8* relative to *Gapdh* transcripts in YUMM3.3 cells 3 d after *Klk8* depletion with four siRNAs. Data show means ±SD of three experiments (One-Way ANOVA). Created in BioRender. Bulliard, M. (2025) *https://BioRender.com/g56m538*. *p < 0.05, **p < 0.01, ***p < 0.001, ****p < 0.0001.

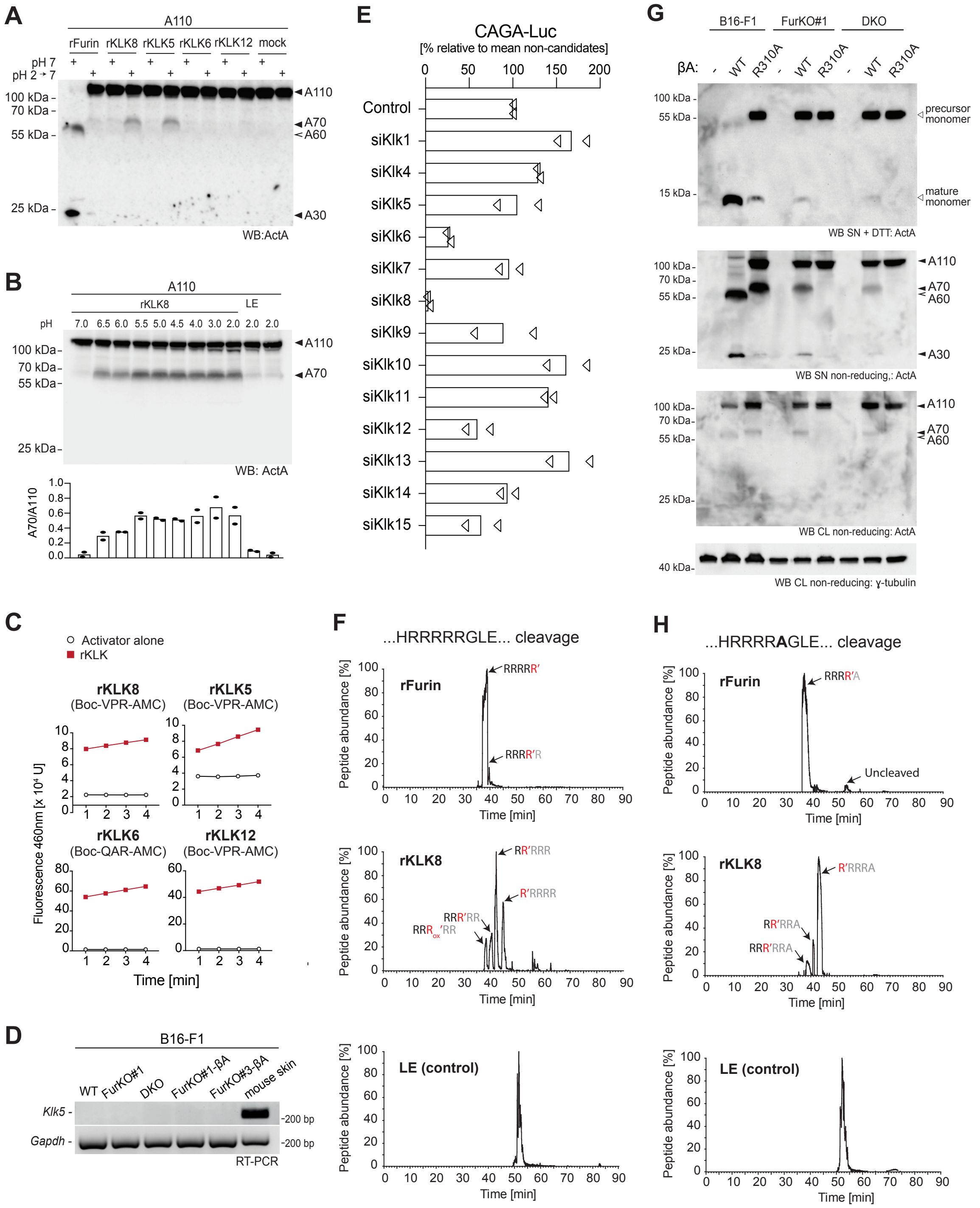

Figure S2. ProActivin-A processing by various kallikreins, and KLK8-mediated cleavage of the wild-type or R310A mutant furin recognition motif in synthetic peptides.

A) Activin-A Western blot of A110 that was transiently acidified to pH 2 for 60 min where indicated, followed by treatment with rFurin or with the indicated recombinant kallikreins (rKLKs) for 5 hrs at pH 7. Data represent two experiments. **B)** Cell-free cleavage of A110 by rKLK8 as in (A), but after incubation of A110 at the indicated pH. Densitometric quantification of A70/A110 ratios from two experiments is shown below. LE, lysyl-endopeptidase. **C)** Fluorescence of the indicated AMC-labelled peptide substrates after incubation with the indicated rKLKs. **D)** RT-PCR analysis of *Klk5* mRNA in parental B16-F1 cells (WT) and in the indicated knockout clones lacking *Furin* alone (FurKO), or both *Furin* and *PSKC7* (DKO), before or after stable transduction with βA. Mouse skin from a 10-week-old female C57BL/6J mouse served as a positive control. Data represent one of two experiments with similar results. **E)** Induction of CAGA-Luc in HepG2.α1-PDX reporter cells that were co-cultured for 24 hrs with

B16F1 FurKO#1-βA cells Values represent the means from two biological replicates after knockdown of the indicated kallikreins relative to the average CAGA-Luc induction by all other SNs ("non-candidates") from the siRNA library screen. **F)** LC-MS/MS spectra of the synthetic 41 amino acid peptide containing the penta-arginine motif of proActivin-A after 5 hrs incubation with rFurin, rKLK8, or alone. Data represent one of two experiments with similar results. The identified cleavage sites are indicated in red. **G)** Differential proteolytic processing of wild-type (WT) versus R310A mutant proActivin-A analyzed after treatment with 0.2 M dithiothreitol (DTT) or under non-reducing conditions by immunoblotting in SNs or cell lysates (CL) of the transiently transfected melanoma cell lines indicated at the top. Open arrowheads mark monomeric forms of mature Activin-A and of its uncleaved precursor migrating with apparent molecular weights of 14 and 56 kDa, respectively. **H)** As in (F), but after mutating the fifth arginine of the RRRRR motif in this peptide to alanine (bold).

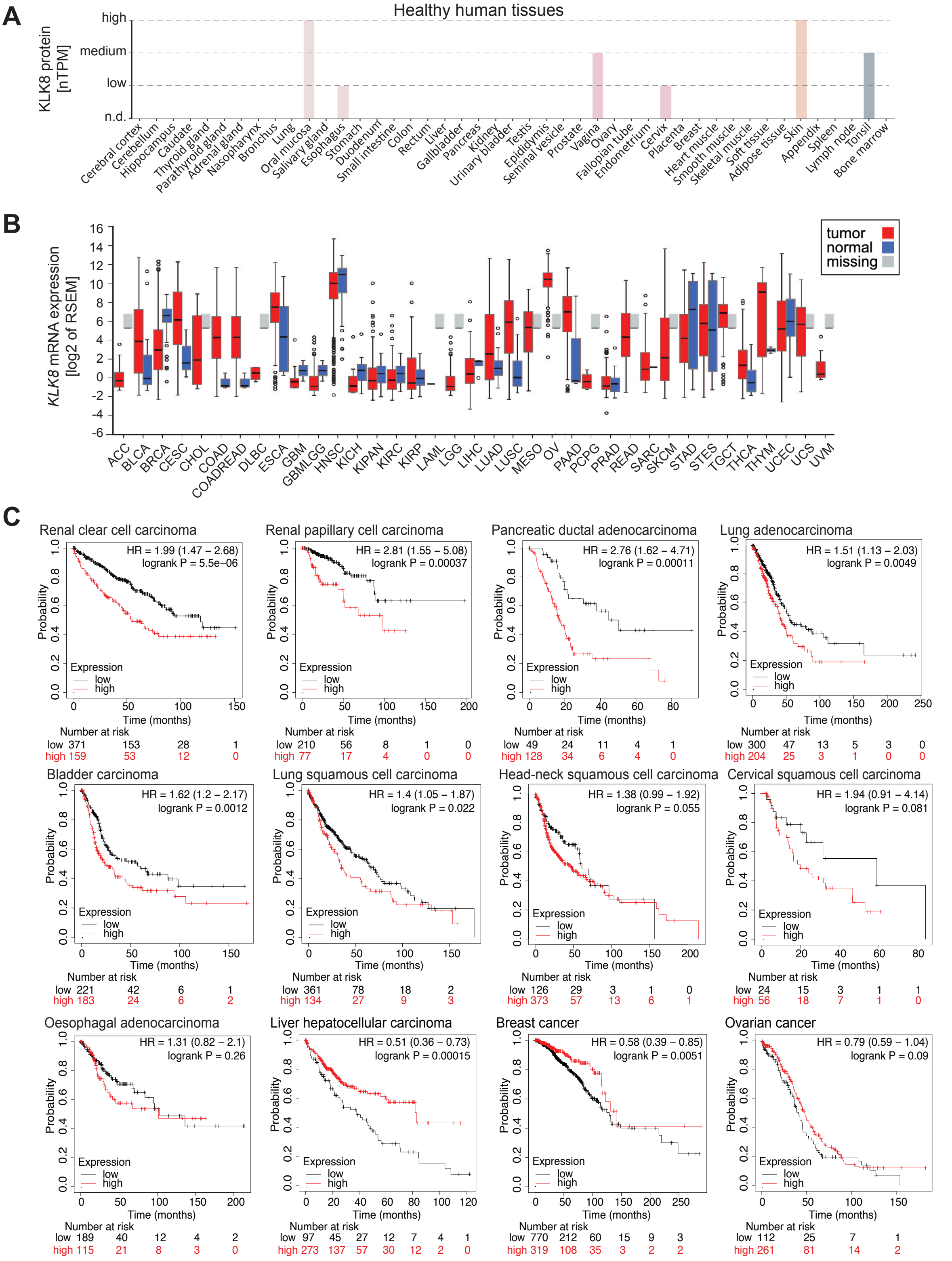

Figure S3. KLK8 is expressed in the skin and upregulated in a subset of tumors compared to healthy control tissues.

A) Analysis of KLK8 protein expression by immunostaining in normal human tissues (The Human Protein Atlas). nTPM, normalized transcripts per million. **B)** *KLK8* mRNA levels across tumor types and healthy control tissues in the TCGA database (FireBrowse). RSEM, RNA-seq by expectation-maximization. **C)** Kaplan Meier plots showing the overall survival of patients expressing low or high levels of *KLK8* mRNA in twelve human cancer types (TCGA database). HR, hazard ratio.

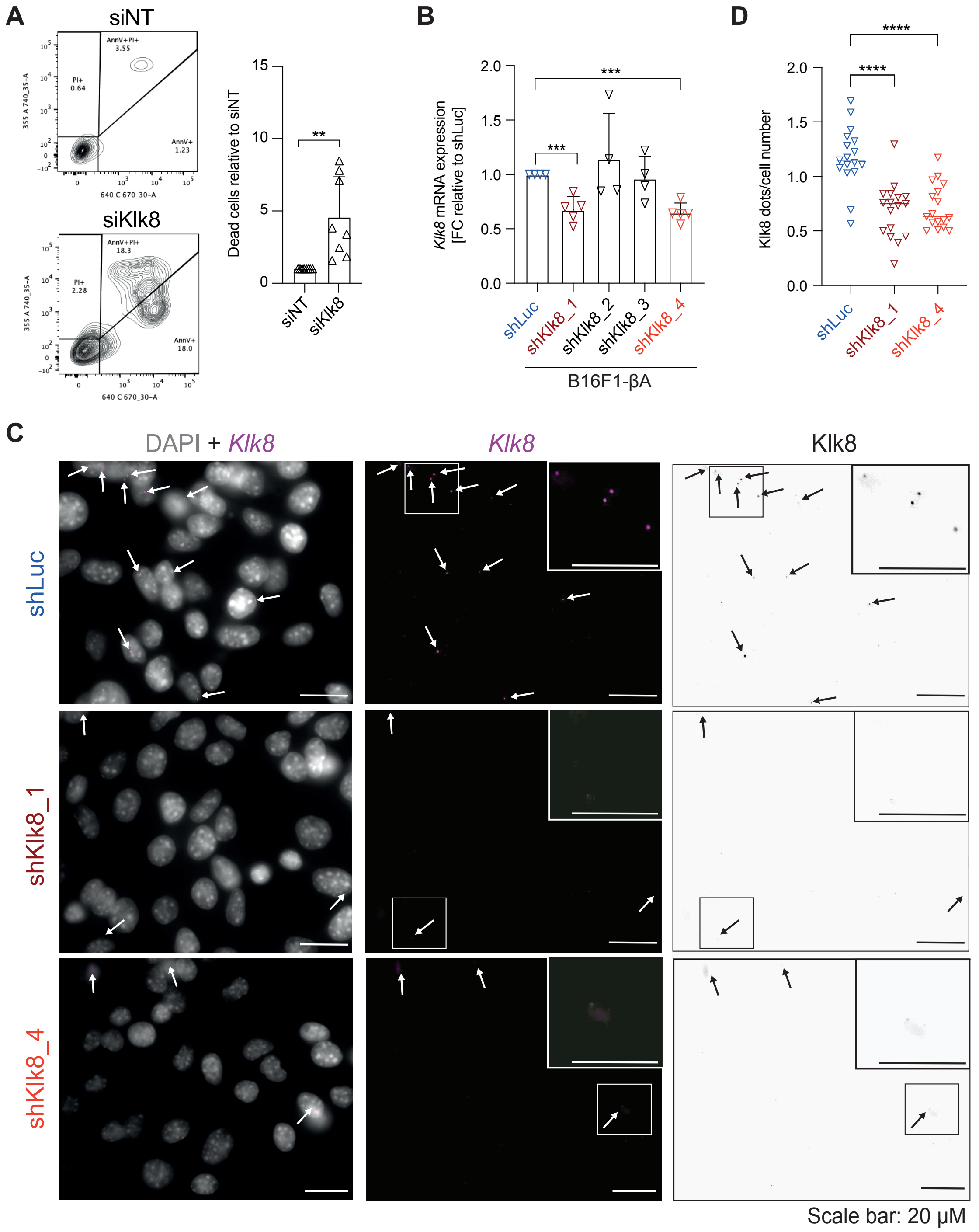

Figure S4. Klk8 knock-down efficiency by four IPTG-inducible shRNAs in B16F1-βA cells.

A) Flow cytometric gating of Annexin V and propidium iodide (PI) stainings used to detect dead B16F1 FurKO#1-βA cells 72 hrs after transfection with siNT or siKlk8. The quantification of double-positive dead cells is shown on the right. Values are normalized to the condition with siNT. Data show the average of eight independent experiments ± SD (Two-sided Welch's t-test). **B)** RT-qPCR analysis of endogenous *Klk8* relative to *Gapdh* mRNA expression in B16F1-βA cells 72 hrs after the induction of different *Klk8* shRNAs by 100 μM IPTG. Data show the average ± SD of four to five experiments, normalized to values in cells transduced with shLuciferase as a negative control (Two-sided Welch's t-test). **C)** RNAscope analysis of *Klk8* mRNA expression in B16F1-βA cells stably transduced with control shRNA (shLuc) or shKlk8_1, or shKlk8_4. Images show representative regions viewed with a 63x objective. Boxed areas are shown at higher magnification in the top right corner. **D)** Quantification of Klk8 dots in the experiment shown in (B). Data show the average of two experiments, with a total of 17 fields analyzed per condition (Two-sided Welch's t-test). **p < 0.01, ***p < 0.001, ****p < 0.0001.

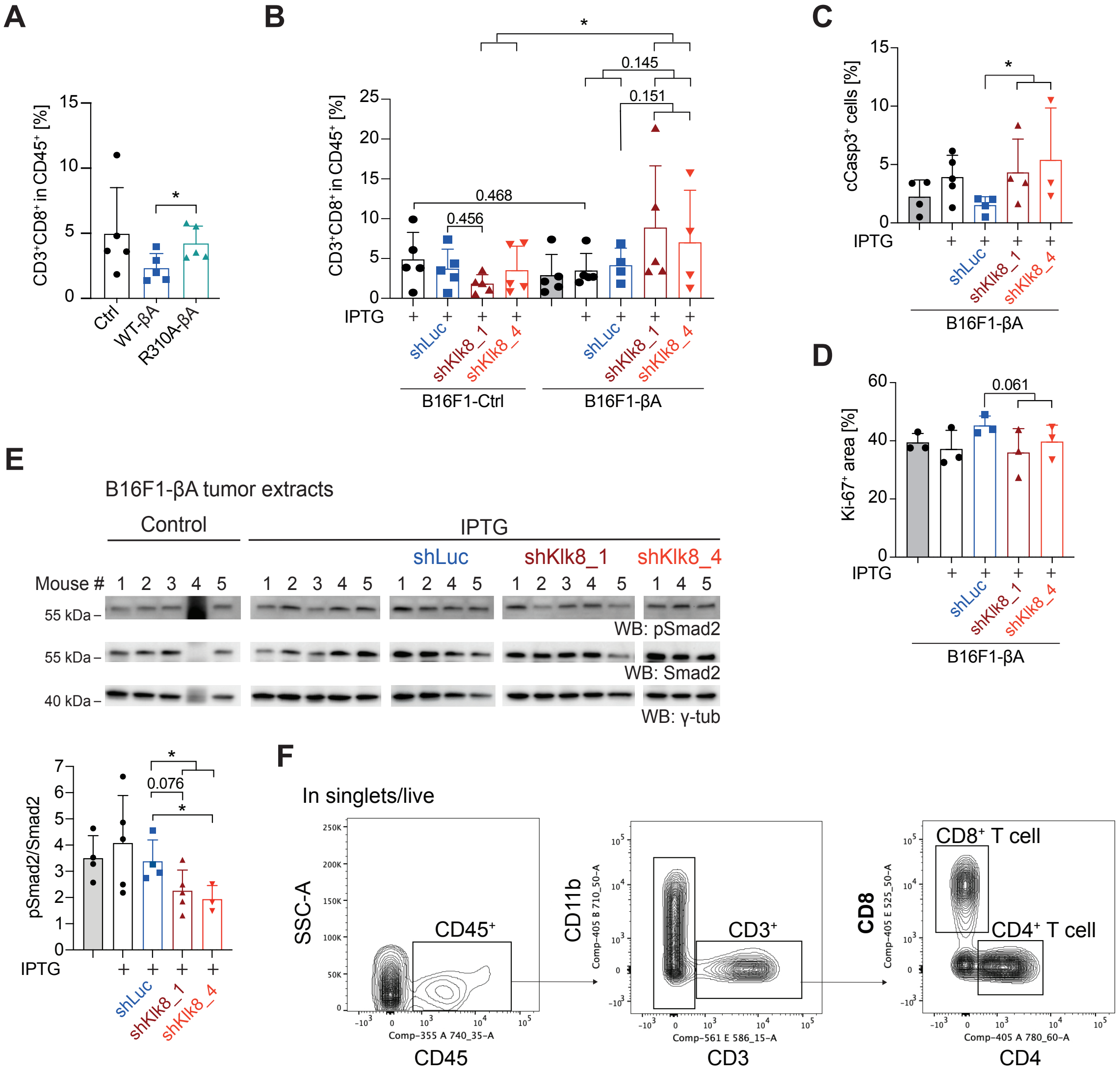

Figure S5. Flow cytometric analysis of tumor infiltration by CD8⁺ T cells.

A) Flow cytometric analysis of the relative proportion of CD8⁺ T cells among the total CD45⁺ leukocytes from B16F1-Ctrl, -βA, or R310A βA-expressing melanoma grafts at the endpoint (n=5 mice per group). Data represent average ± SD (Two-sided Mann-Whitney t-test). **B)** Proportion of tumor-infiltrating CD8⁺ T cells in B16F1-Ctrl (left) and -βA (right) tumors with no shRNA, shLuc, shKlk8_1, or shKlk8_4 that were grafted in hosts receiving IPTG where indicated (n=4 to 10 mice per group; Two-sided Welch's t-tests). **C)** Quantification of cCasp3⁺ cells and of **D)** Ki-67⁺ areas in sections of tumors (n≥3 per group). Data represent average ± SD (Two-sided Welch's t-tests). **E)** pSmad2, Smad2, and γ-tubulin Western blots of extracts of B16F1-βA tumors from the cohort of tumor-bearing mice in figure 5B. The pSmad2/Smad2 quantification is shown below (Two-sided Welch's t-tests). **F)** FACS gating strategy used to quantify CD8⁺ T cells in figure S5A, B. *$p < 0.05$.
