# Supplementary material for: Kallikrein-8 mediates furin-independent Activin-A precursor processing to stimulate tumor growth in melanoma
Source: Nat Commun. 2025 Mar 10;16:2354. doi: 10.1038/s41467-025-57661-5 (PMC11893775; doi:10.1038/s41467-025-57661-5)
Supplement: Supplementary file 2 — Description of Additional Supplementary Information [file 41467_2025_57661_MOESM2_ESM.pdf]

Description of Additional Supplementary Files

Supplementary Data 1 – Peptide fragments generated by rFurin versus rKLK8 treatment
Supplementary Data 2 – KLK8 status of primary and metastatic human melanoma
