# Supplementary material for: Kallikrein-8 mediates furin-independent Activin-A precursor processing to stimulate tumor growth in melanoma
Source: Nat Commun. 2025 Mar 10;16:2354. doi: 10.1038/s41467-025-57661-5 (PMC11893775; doi:10.1038/s41467-025-57661-5)
Supplement: Supplementary file 5 — Reporting Summary [file 41467_2025_57661_MOESM5_ESM.pdf]

nature portfolio

Corresponding author(s): Daniel B. Constam

Last updated by author(s): 17.12.2024

Reporting Summary

Nature Portfolio wishes to improve the reproducibility of the work that we publish. This form provides structure for consistency and transparency in reporting. For further information on Nature Portfolio policies, see our Editorial Policies and the Editorial Policy Checklist.

Statistics

For all statistical analyses, confirm that the following items are present in the figure legend, table legend, main text, or Methods section.

n/a	Confirmed	
[ ]	[x]	The exact sample size (*n*) for each experimental group/condition, given as a discrete number and unit of measurement
[ ]	[x]	A statement on whether measurements were taken from distinct samples or whether the same sample was measured repeatedly
[ ]	[x]	The statistical test(s) used AND whether they are one- or two-sided<br>*Only common tests should be described solely by name; describe more complex techniques in the Methods section.*
[x]	[ ]	A description of all covariates tested
[ ]	[x]	A description of any assumptions or corrections, such as tests of normality and adjustment for multiple comparisons
[ ]	[x]	A full description of the statistical parameters including central tendency (e.g. means) or other basic estimates (e.g. regression coefficient) AND variation (e.g. standard deviation) or associated estimates of uncertainty (e.g. confidence intervals)
[ ]	[x]	For null hypothesis testing, the test statistic (e.g. *F*, *t*, *r*) with confidence intervals, effect sizes, degrees of freedom and *P* value noted<br>*Give P values as exact values whenever suitable.*
[x]	[ ]	For Bayesian analysis, information on the choice of priors and Markov chain Monte Carlo settings
[x]	[ ]	For hierarchical and complex designs, identification of the appropriate level for tests and full reporting of outcomes
[x]	[ ]	Estimates of effect sizes (e.g. Cohen's *d*, Pearson's *r*), indicating how they were calculated

Our web collection on statistics for biologists contains articles on many of the points above.

Software and code

Policy information about availability of computer code

Data collection	TCGA Melanoma (SKCM) gene expression RNAseq (HiSeqV2), clinical and survival data were downloaded from the UCSC Xena Hub using R free statistical software (v. 4.3.0) and the UCSCXenaTools package (v. 1.4.7).
Data analysis	Data were analyzed with R free statistical software (v. 4.3.0), survival (v. 3.5.0), survminer (v. 0.4.9) and ggpubr (v. 0.6.0) packages.

For manuscripts utilizing custom algorithms or software that are central to the research but not yet described in published literature, software must be made available to editors and reviewers. We strongly encourage code deposition in a community repository (e.g. GitHub). See the Nature Portfolio guidelines for submitting code & software for further information.

Data

Policy information about availability of data

All manuscripts must include a data availability statement. This statement should provide the following information, where applicable:

- Accession codes, unique identifiers, or web links for publicly available datasets
- A description of any restrictions on data availability
- For clinical datasets or third party data, please ensure that the statement adheres to our policy

All the data reported in this study, including the source data behind the graphs and uncropped images of all Western blots, are available within the paper, or in the Supplementary Information and Source Data File online. Request for resources and reagents should be sent to and will be answered by the lead contact Prof. Daniel B. Constam (daniel.constam@epfl.ch).

Research involving human participants, their data, or biological material

Policy information about studies with human participants or human data. See also policy information about sex, gender (identity/presentation), and sexual orientation and race, ethnicity and racism.

Reporting on sex and gender	n.a.
Reporting on race, ethnicity, or other socially relevant groupings	n.a.
Population characteristics	n.a.
Recruitment	n.a.
Ethics oversight	n.a.

Note that full information on the approval of the study protocol must also be provided in the manuscript.

Field-specific reporting

Please select the one below that is the best fit for your research. If you are not sure, read the appropriate sections before making your selection.

☒ Life sciences ☐ Behavioural & social sciences ☐ Ecological, evolutionary & environmental sciences

For a reference copy of the document with all sections, see nature.com/documents/nr-reporting-summary-flat.pdf

Life sciences study design

All studies must disclose on these points even when the disclosure is negative.

Sample size	The sizes of animal cohorts were approved by our animal experimentation ethics committee based on the past experience of effect sizes in this tumor grafting model. Luciferase reporter assays were repeated independently at least twice using each time three technical replicates per sample in keeping with standard practice in the field for this kind of assay, and based on our own experience over the past 10 years with the reporter cell lines used in this study.
Data exclusions	One tumor from the WT-BA, one from the WT-BA shLuc, and one from the WT-BA shKlk8_1 group (Fig. 5B) did not grow so these three tumors were excluded from the growth curves. One tumor from FurKO#2-BA IPTG and one from the FurKO#2-BA shLuc IPTG (Fig. 5E) did not grow and were thus excluded. In the figure 5G only 4 tumors represent the Activin-A forms in these two groups.
Replication	All experiments were repeated independently as indicated in the figure legends, and with individual values depicted as scatter plots to indicate their distribution.
Randomization	All samples and animals were allocated randomly.
Blinding	Where possible, treated groups were analyzed without knowing the treatment or their genotypes (e.g., for IF staining of tumor sections, analysis of colony growth in soft agar, RNA scope analysis, etc.). By contrast, to analyze tumor-bearing mice we did not try to blind the investigators because of overt differences between treated groups and controls, and becasue IPTG in the drinking water is readily recognized by its smell.

Reporting for specific materials, systems and methods

We require information from authors about some types of materials, experimental systems and methods used in many studies. Here, indicate whether each material, system or method listed is relevant to your study. If you are not sure if a list item applies to your research, read the appropriate section before selecting a response.

Materials & experimental systems

n/a	Involved in the study	
☐	☒	Antibodies
☐	☒	Eukaryotic cell lines
☒	☐	Palaeontology and archaeology
☐	☒	Animals and other organisms
☒	☐	Clinical data
☒	☐	Dual use research of concern
☒	☐	Plants

Methods

n/a	Involved in the study	
☒	☐	ChIP-seq
☐	☒	Flow cytometry
☒	☐	MRI-based neuroimaging

Antibodies

Antibodies used	All antibodies used for FACS analysis were diluted 1:200 and validated for this application by the manufacturers and by references cited on their websites, including CD11b BV711 (M1/70, cat. no. 101241), CD3 PE (clone 145-2C11, cat. no. 100308), and CD4 BV785 (clone RM4-5, cat. no. 100552) from BioLegend, and CD45.2 BUV737 (clone 104, cat. no. 564880) and CD8 BV510 (clone 53-6.7, cat. no. 563068) from BD Horizon.<br>Antibodies to detect Activin-A by Western blot analysis were from Abcam (1:500, Ab89307) and from R&D Systems (0.5 µg/ml, BAM3381). Their specificity was validated by the manufacturers and by our comparisons to control samples lacking Activin-A in this and previous studies from our own lab.<br>Antibodies against Ki-67 (Thermo Fisher Scientific, MA5-14520, 1:100) or cleaved caspase 3 (Cell Signaling, cat. no. 9661, 1:100) were validated by the manufacturers and using negative control immunostainings by secondary anti-rabbit-Alexa568 antibodies alone (Thermo Fisher Scientific, cat. no. A10042, 1:800).
Validation	The validation methods are mentioned above together with the description of the antibodies.

Eukaryotic cell lines

Policy information about cell lines and Sex and Gender in Research

Cell line source(s)	No primary cell lines or cells from human participants were used.
Authentication	The cell lines used in this study were purchased from the American Type Culture Collection (ATCC), including HepG2 (cat. no. HB-8065), HEK293T (cat. no. CRL-3216), B16-F1 (cat. no. CRL-6323), SK-MEL-1 (cat. no. HTB-67), SK-MEL-3 (cat. no. HTB-69), SH-4 (cat. no. CRL-7724) and C32TG cells (cat. no. CRL-1579). Although none of these cell lines were authenticated genetically, they can be readily distinguished from one another based on their characteristics such as cell shapes, sizes, and/or pigmentation. YUMM3.3 cells were a gift of Dr. Anna Obenauf (IMP, Vienna). SCC-12 and SCC-13 cells were provided by Dr. Freddy Radtke, EPFL, Lausanbe). C8161 and C81-61 cells were obtained from Dr. Mary Hendrix (Northwestern Univ., Chicago). Me204, Me275, Me343, and T618, T640, and T672A cells were obtained from Dr. Donata Rimoldi (Ludwig Cancer Institute, Lausanne).
Mycoplasma contamination	All cell lines were cultured for maximally 6-8 weeks after de-freezing, and only after testing negative for mycoplasma infections (Mycospy, Biontex, Munich, DE).
Commonly misidentified lines<br>(See ICLAC register)	-

Animals and other research organisms

Policy information about studies involving animals; ARRIVE guidelines recommended for reporting animal research, and Sex and Gender in Research

Laboratory animals	B16-F1 mouse melanoma cells were grafted into female C57/Bl6 mice.
Wild animals	n.a.
Reporting on sex	Analysis was limited to female mice since male hosts would have to be housed individually to ensure that tumor grafts are not damaged by infighting. Furthermore, while women have a lower risk of melanoma metastasis and female mice show slightly superior anti-tumor immunity against the highly metastatic B16-F10 subline (Dakup et al. Pigment Cell & Melanoma Research 2022 35:268-279), tumor growth of the original non-metastatic B16 mouse melanoma cells is comparable in male and female hosts (Simon & Ershler et al., J Natl Cancer Inst 1985; 74:1085-8).
Field-collected samples	n.a.
Ethics oversight	The study was was conducted in accordance with the local legislation and institutional requirements with the approval of the veterinary administration of the canton of Vaud, Chemin des Boveresses 155, 1066 Epalinges.

Note that full information on the approval of the study protocol must also be provided in the manuscript.

Plants

Seed stocks: n.a.

Novel plant genotypes: n.a.

Authentication: n.a.

Flow Cytometry

Plots

Confirm that:

- [x] The axis labels state the marker and fluorochrome used (e.g. CD4-FITC).
- [x] The axis scales are clearly visible. Include numbers along axes only for bottom left plot of group (a 'group' is an analysis of identical markers).
- [x] All plots are contour plots with outliers or pseudocolor plots.
- [ ] A numerical value for number of cells or percentage (with statistics) is provided.

Methodology

Sample preparation	Tumors were dissected, minced using rounded scissors, and digested in Dnase-I (0.02 mg/mL) and collagenase (1 mg/mL) in RPMI using a gentleMACS Octo Dissociator. Red blood cells were lysed using PharmLyse buffer. After washing in PBS, 1-5 x 106 of cells were used for staining. Cells were incubated with mouse FcR blocking solution (1:200) and with Live/Dead fixable blue dead cell stain (1:1000) for 30 min. After washing, cell surface markers were labeled with specific antibodies for 45 min in FACS buffer (2 % FBS, 2 mM EDTA in PBS). After the staining, cells were collected in FACS buffer, and data were acquired using an LSR Fortessa cytometer.
Instrument	LSR Fortessa cytometer (Becton Dickinson, Franklin Lakes, NJ, USA).
Software	FlowJo
Cell population abundance	The CD45+ population represents around 10% of the viable cells. Whitin this population, around 20% of the cells are CD3+. Around 10% of the CD3+ cells are CD4+, and around 5% CD8+.
Gating strategy	The lymphocytes were selected based on SSC-A/FSC-A. Then single cells were selected using SSC-A/SSC-H, and a second single cell selection was performed using FSC-A/FSC-H. The live cells were then selected based on the live/dead staining. CD45+ cells are then gated, and then the CD3 population, and from the CD3+ cells, CD4 and CD8 populations are gated.

- [x] Tick this box to confirm that a figure exemplifying the gating strategy is provided in the Supplementary Information.
