# Supplementary material for: Kallikrein-8 mediates furin-independent Activin-A precursor processing to stimulate tumor growth in melanoma
Source: Nat Commun. 2025 Mar 10;16:2354. doi: 10.1038/s41467-025-57661-5 (PMC11893775; doi:10.1038/s41467-025-57661-5)

1B

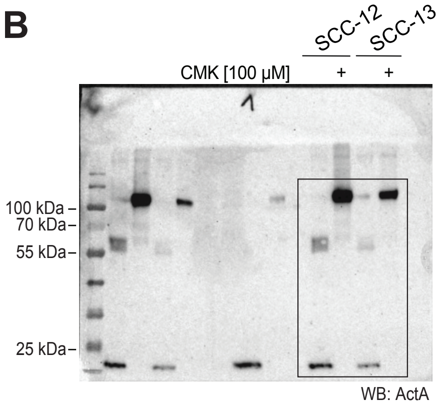
SCC-12
SCC-13
CMK [100 µM]
+
+
100 kDa
70 kDa
55 kDa
25 kDa
WB: ActA

1C

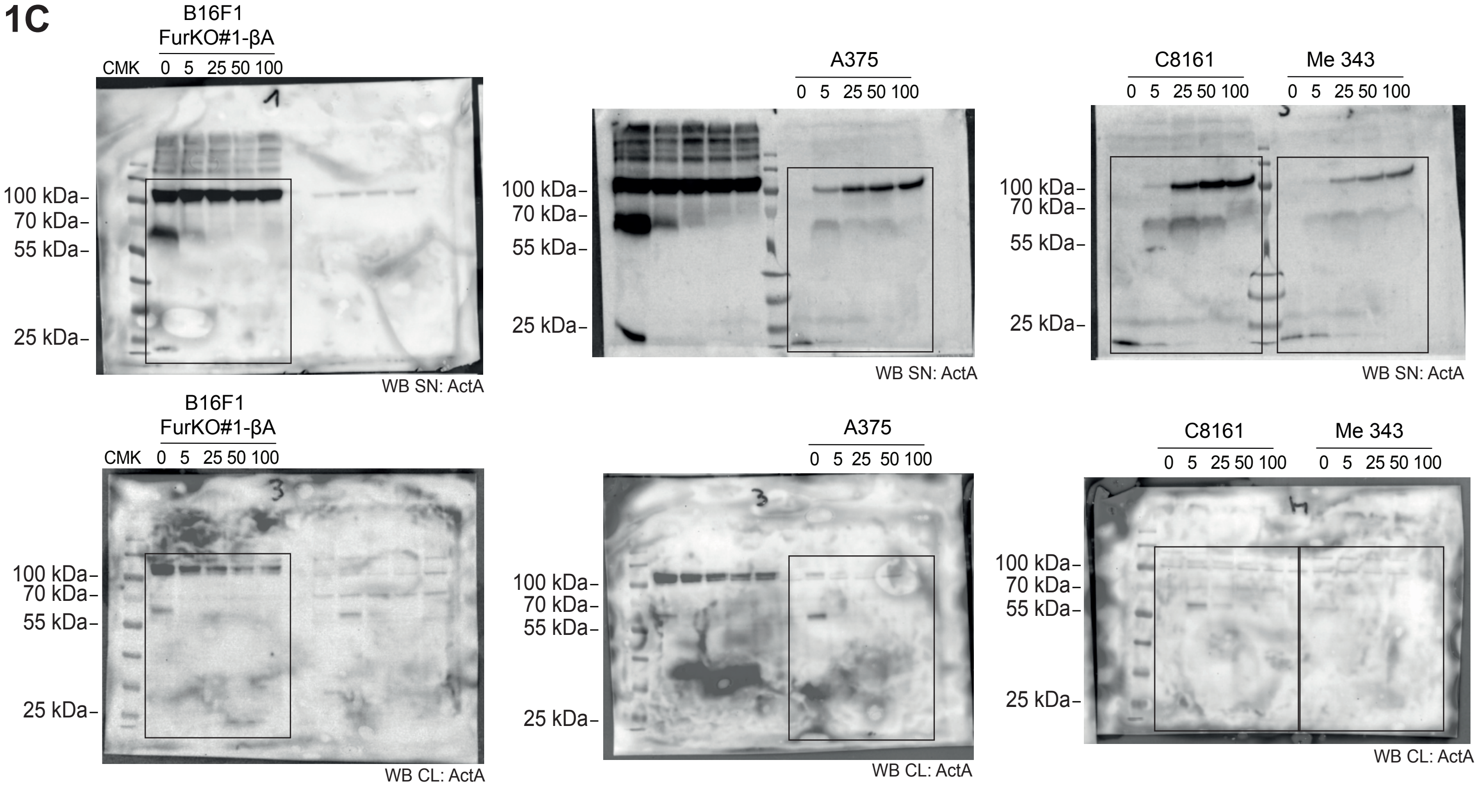
B16F1
FurKO#1-βA
CMK 0 5 25 50 100
100 kDa
70 kDa
55 kDa
25 kDa
WB SN: ActA
A375
0 5 25 50 100
100 kDa
70 kDa
55 kDa
25 kDa
WB SN: ActA
C8161
0 5 25 50 100
Me 343
0 5 25 50 100
100 kDa
70 kDa
55 kDa
25 kDa
WB SN: ActA
B16F1
FurKO#1-βA
CMK 0 5 25 50 100
100 kDa
70 kDa
55 kDa
25 kDa
WB CL: ActA
A375
0 5 25 50 100
100 kDa
70 kDa
55 kDa
25 kDa
WB CL: ActA
C8161
0 5 25 50 100
Me 343
0 5 25 50 100
100 kDa
70 kDa
55 kDa
25 kDa
WB CL: ActA

1E

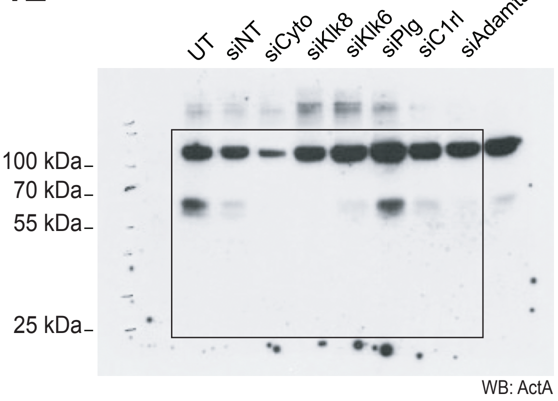
UT
siNT
siCyto
siKlk8
siKlk6
siPlg
siC1rl
siAdamts10
100 kDa
70 kDa
55 kDa
25 kDa
WB: ActA

1F

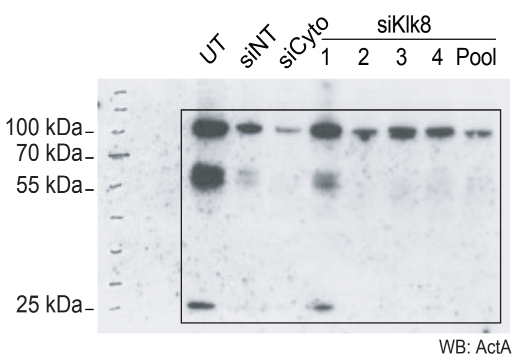
UT
siNT
siCyto
siKlk8
1 2 3 4 Pool
100 kDa
70 kDa
55 kDa
25 kDa
WB: ActA

2A

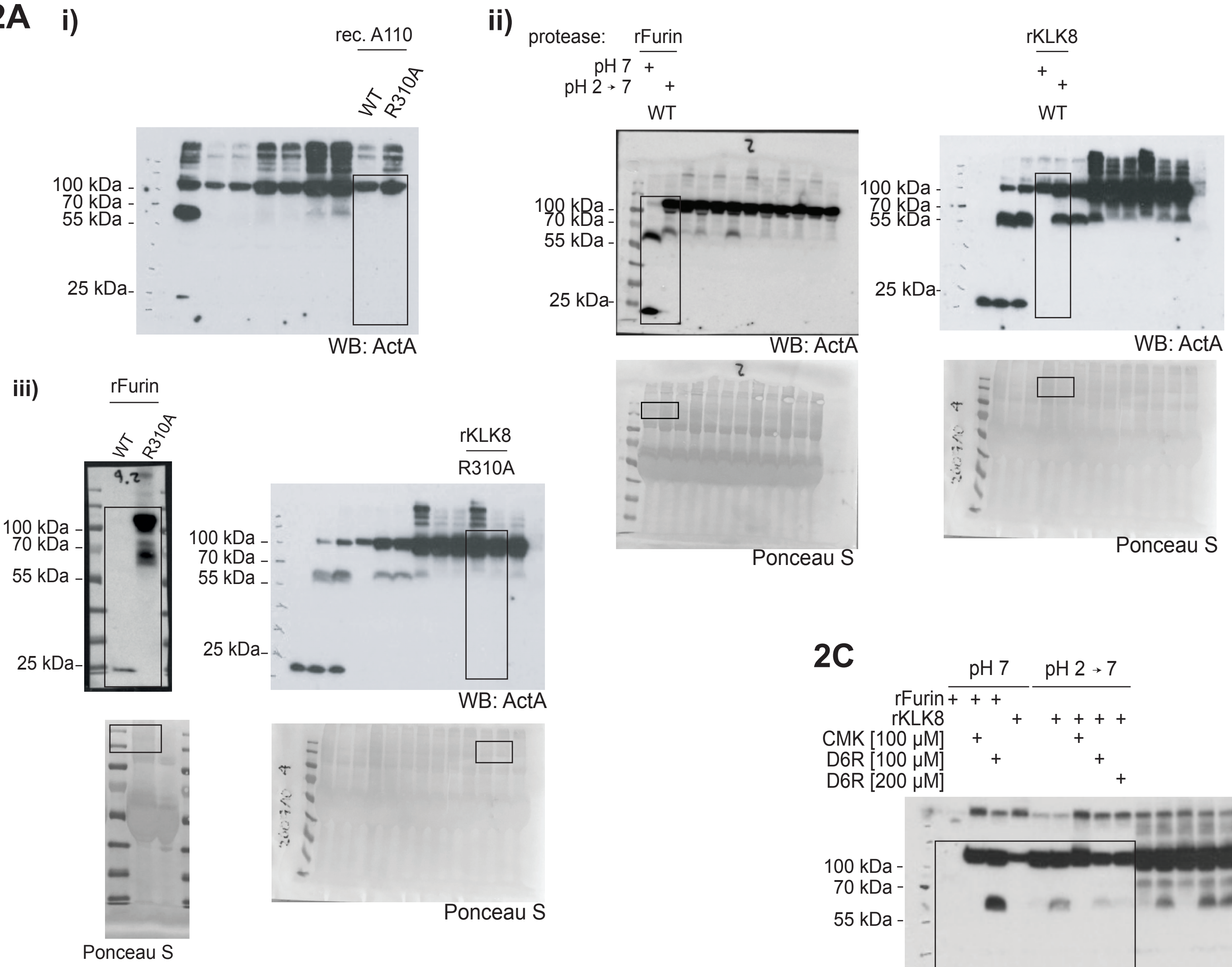

i)
rec. A110
WT
R310A
100 kDa
70 kDa
55 kDa
25 kDa
WB: ActA
ii)
protease:
rFurin
pH 7
pH 2 → 7
WT
100 kDa
70 kDa
55 kDa
25 kDa
WB: ActA
Ponceau S
rKLK8
WT
100 kDa
70 kDa
55 kDa
25 kDa
WB: ActA
Ponceau S
iii)
rFurin
WT
R310A
100 kDa
70 kDa
55 kDa
25 kDa
Ponceau S
rKLK8
R310A
100 kDa
70 kDa
55 kDa
25 kDa
WB: ActA
Ponceau S

2C

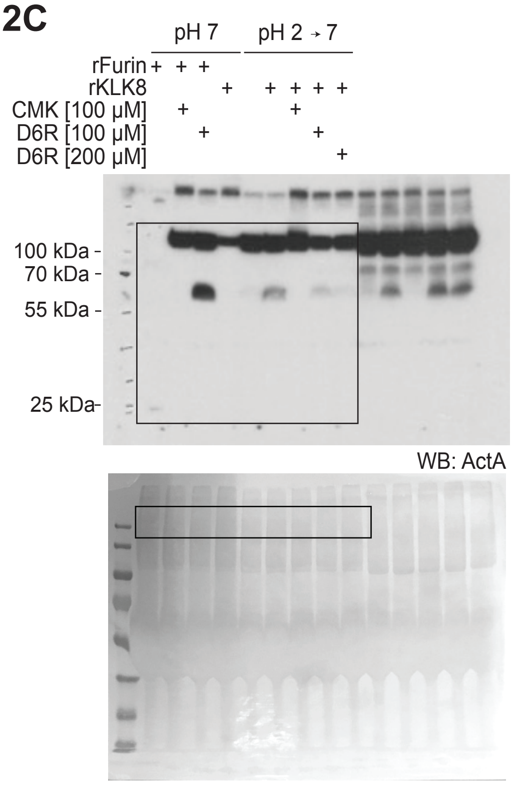

pH 7
pH 2 → 7
rFurin
rKLK8
CMK [100 μM]
D6R [100 μM]
D6R [200 μM]
100 kDa
70 kDa
55 kDa
25 kDa
WB: ActA

2E

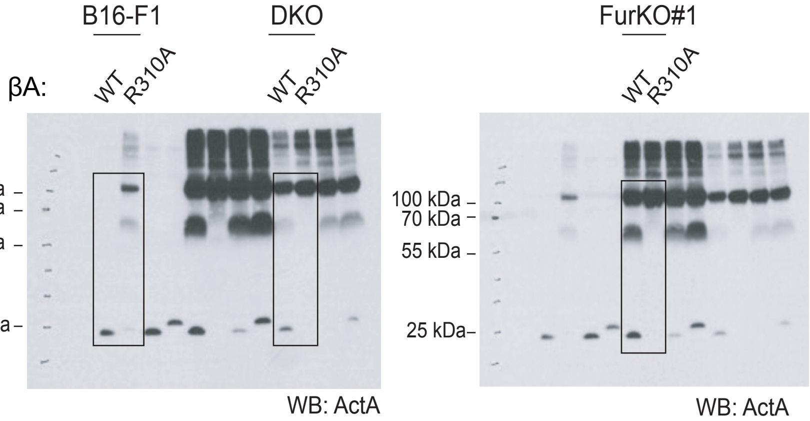

B16-F1
DKO
FurKO#1
βA:
WT
R310A
100 kDa
70 kDa
55 kDa
25 kDa
WB: ActA

2G

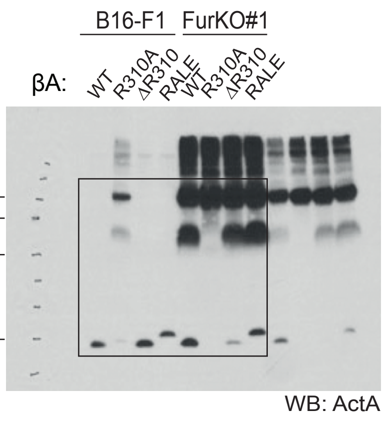

B16-F1
FurKO#1
βA:
WT
R310A
ΔR310
RALE
100 kDa
70 kDa
55 kDa
25 kDa
WB: ActA

2I

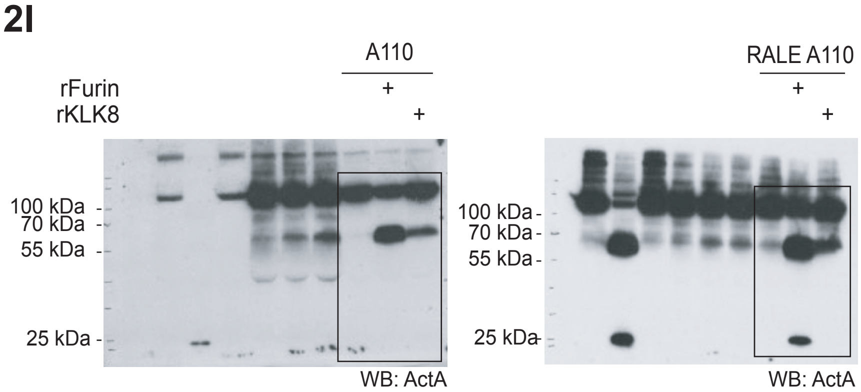

A110
RALE A110
rFurin
rKLK8
100 kDa
70 kDa
55 kDa
25 kDa
WB: ActA

3E

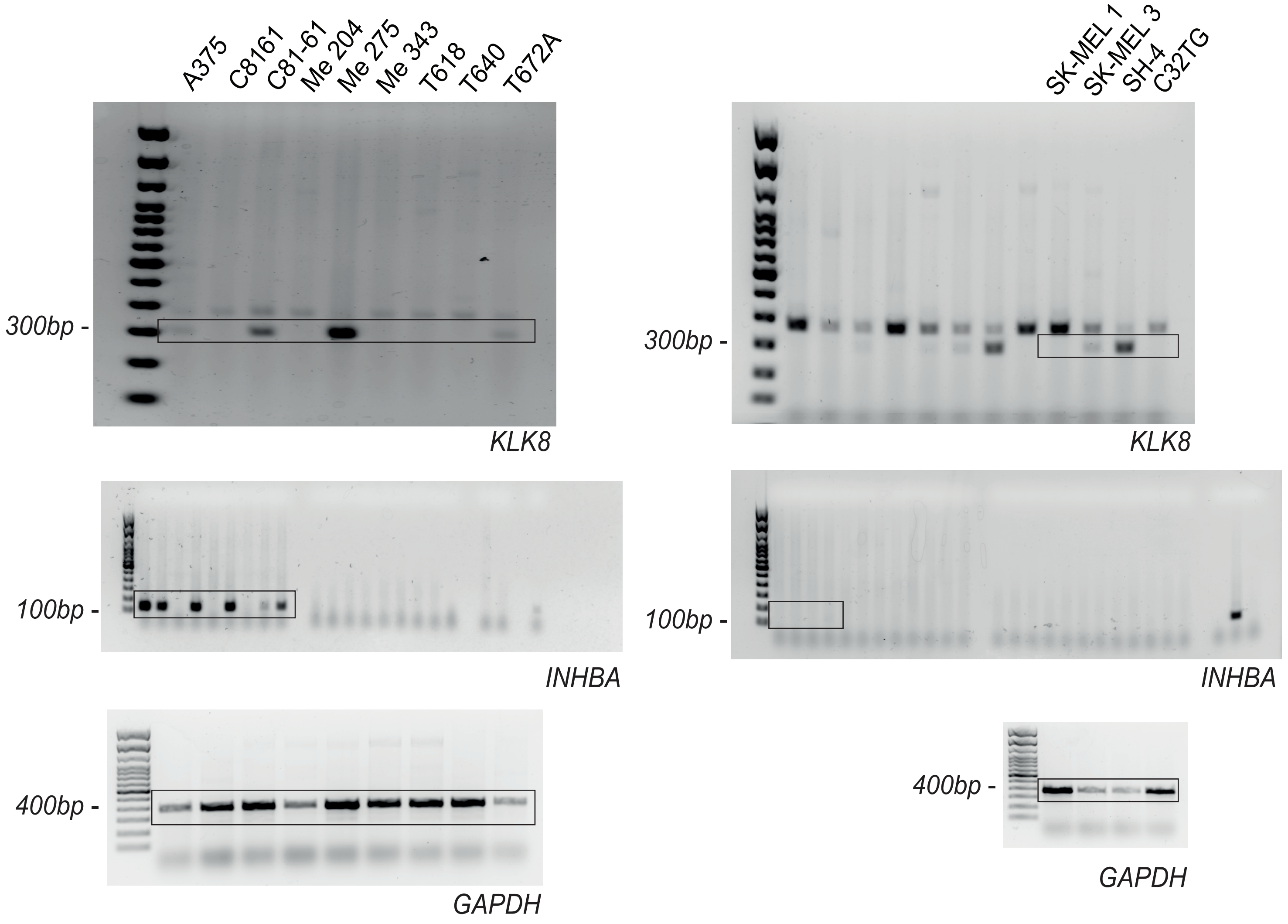
A375
C8161
C81-61
Me 204
Me 275
Me 343
T618
T640
T672A
300bp -
KLK8
SK-MEL 1
SK-MEL 3
SH-4
C32TG
300bp -
KLK8
100bp -
INHBA
100bp -
INHBA
400bp -
GAPDH
400bp -
GAPDH

3F

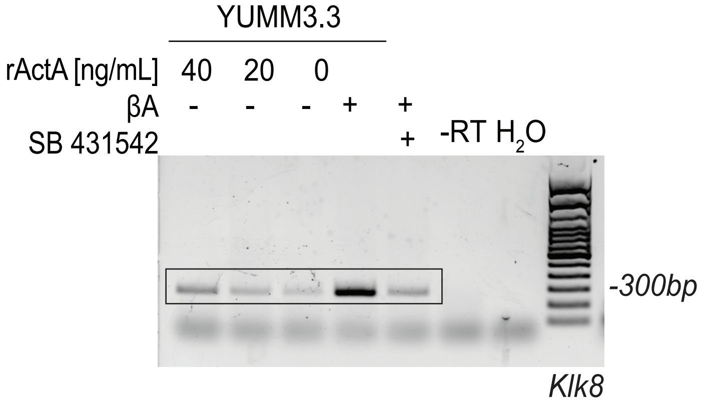
YUMM3.3
rActA [ng/mL] 40 20 0
βA - - - + +
SB 431542 +
-RT H2O
-300bp
Klk8

4C

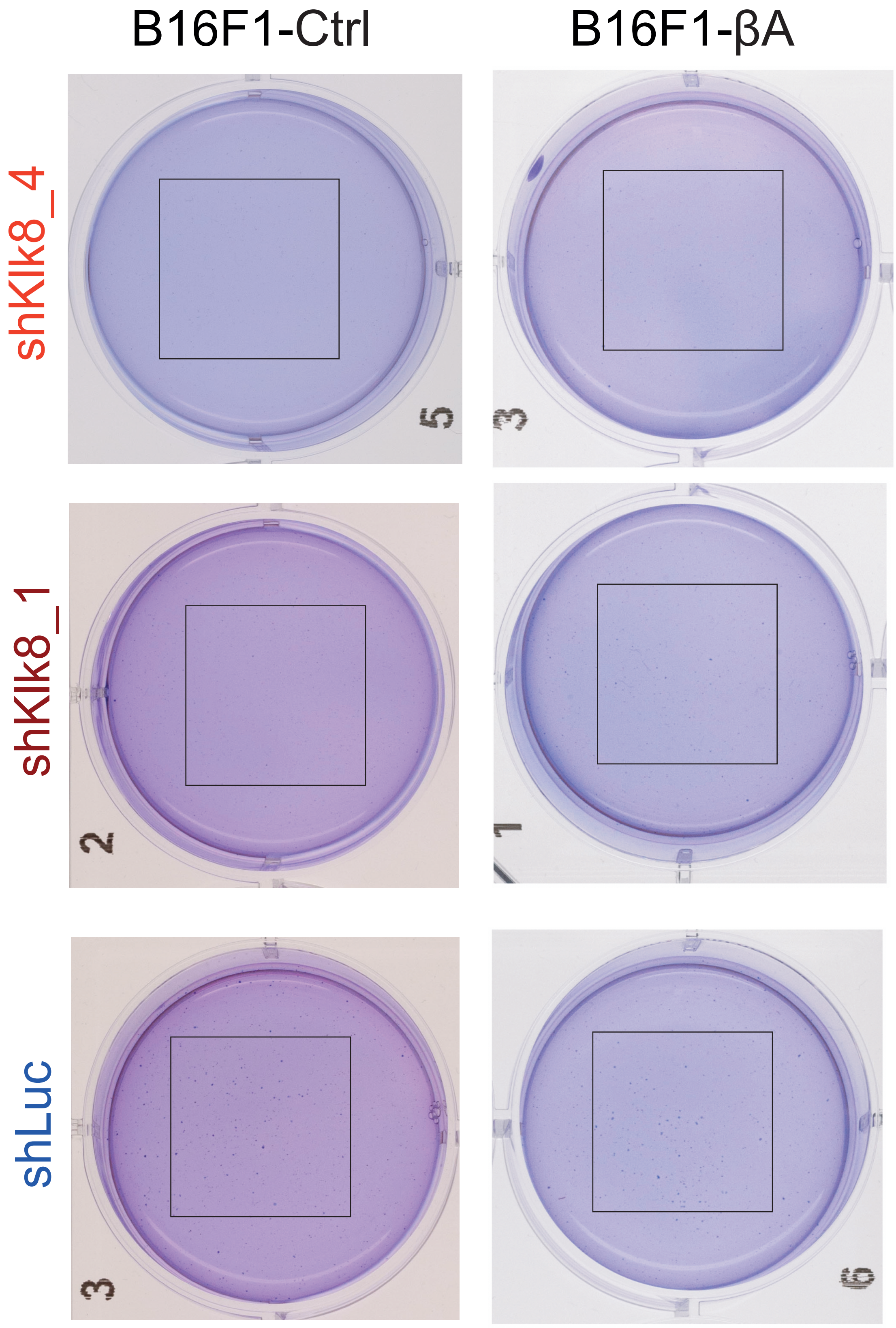

5C

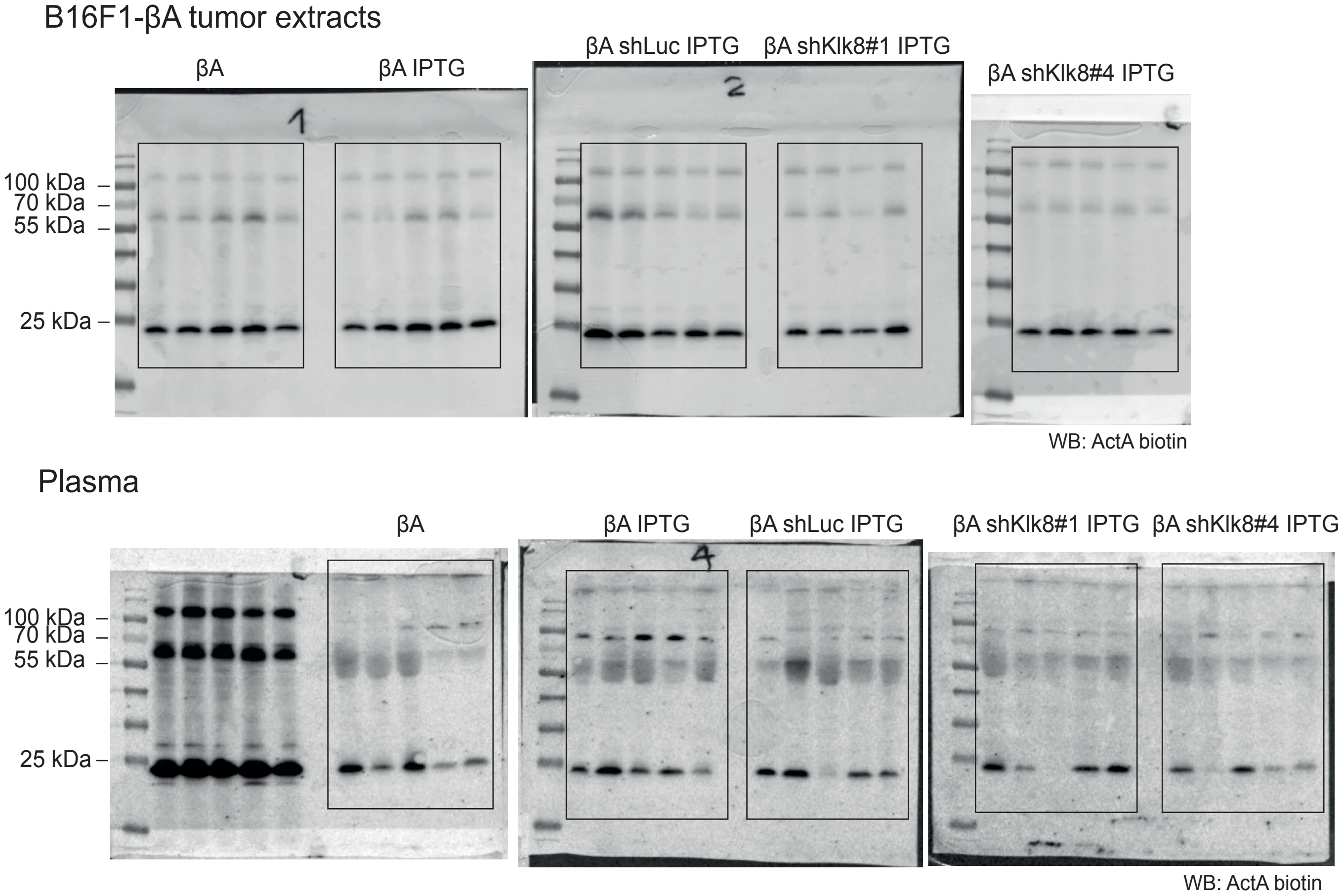
B16F1-βA tumor extracts
βA
βA IPTG
βA shLuc IPTG
βA shKlk8#1 IPTG
βA shKlk8#4 IPTG
100 kDa
70 kDa
55 kDa
25 kDa
WB: ActA biotin
Plasma
βA
βA IPTG
βA shLuc IPTG
βA shKlk8#1 IPTG
βA shKlk8#4 IPTG
100 kDa
70 kDa
55 kDa
25 kDa
WB: ActA biotin

5F

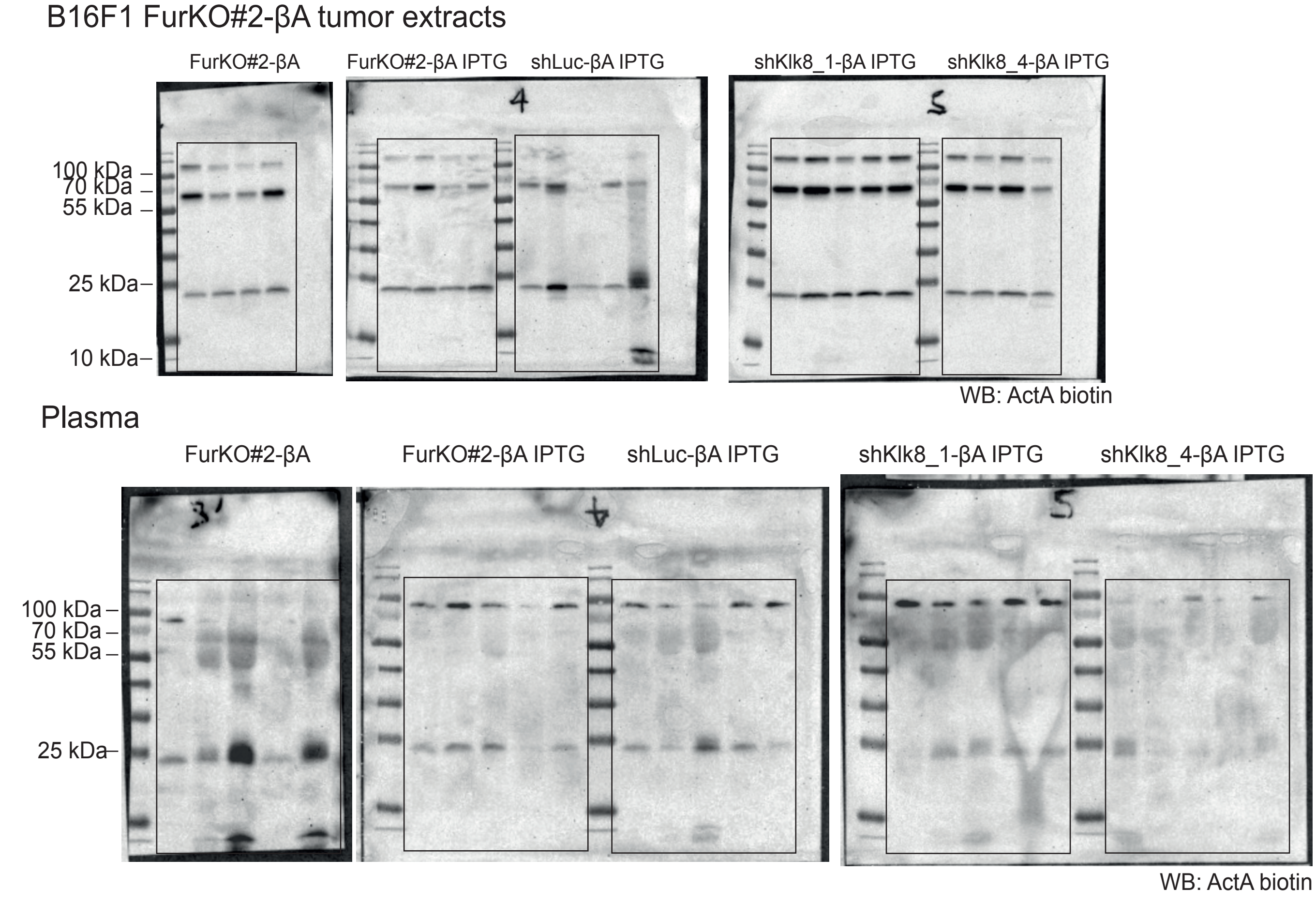
B16F1 FurKO#2-βA tumor extracts
FurKO#2-βA
FurKO#2-βA IPTG
shLuc-βA IPTG
shKlk8_1-βA IPTG
shKlk8_4-βA IPTG
100 kDa
70 kDa
55 kDa
25 kDa
10 kDa
WB: ActA biotin
Plasma
FurKO#2-βA
FurKO#2-βA IPTG
shLuc-βA IPTG
shKlk8_1-βA IPTG
shKlk8_4-βA IPTG
100 kDa
70 kDa
55 kDa
25 kDa
WB: ActA biotin

6C

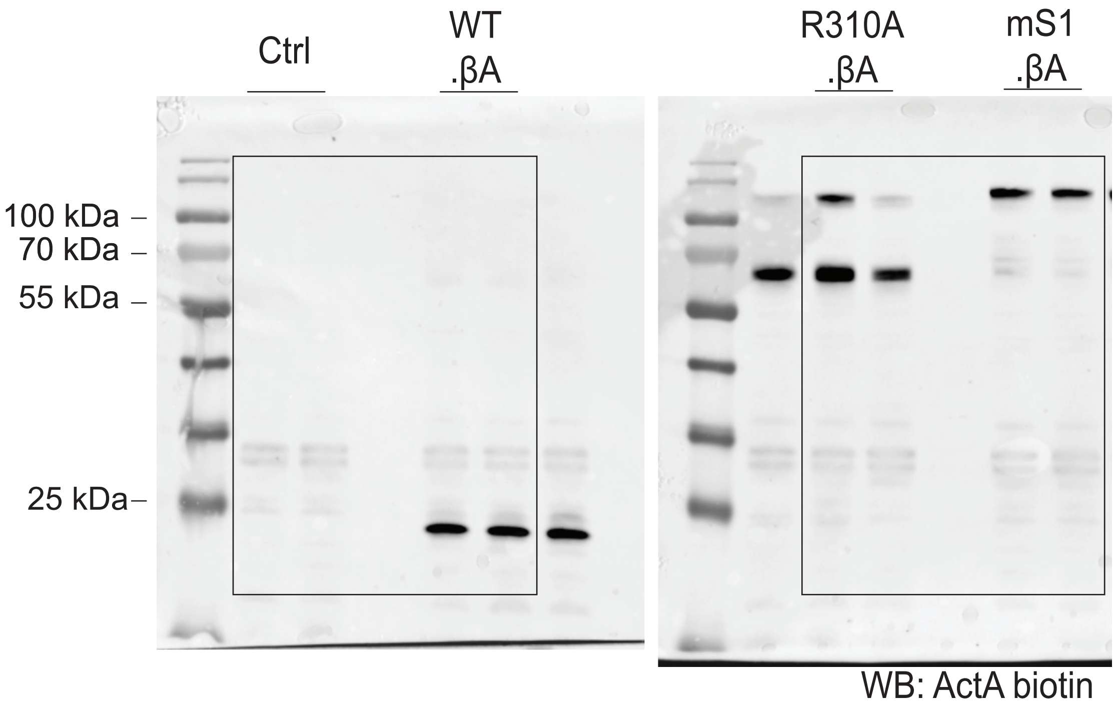
Ctrl
WT .βA
R310A .βA
mS1 .βA
100 kDa
70 kDa
55 kDa
25 kDa
WB: ActA biotin

6F

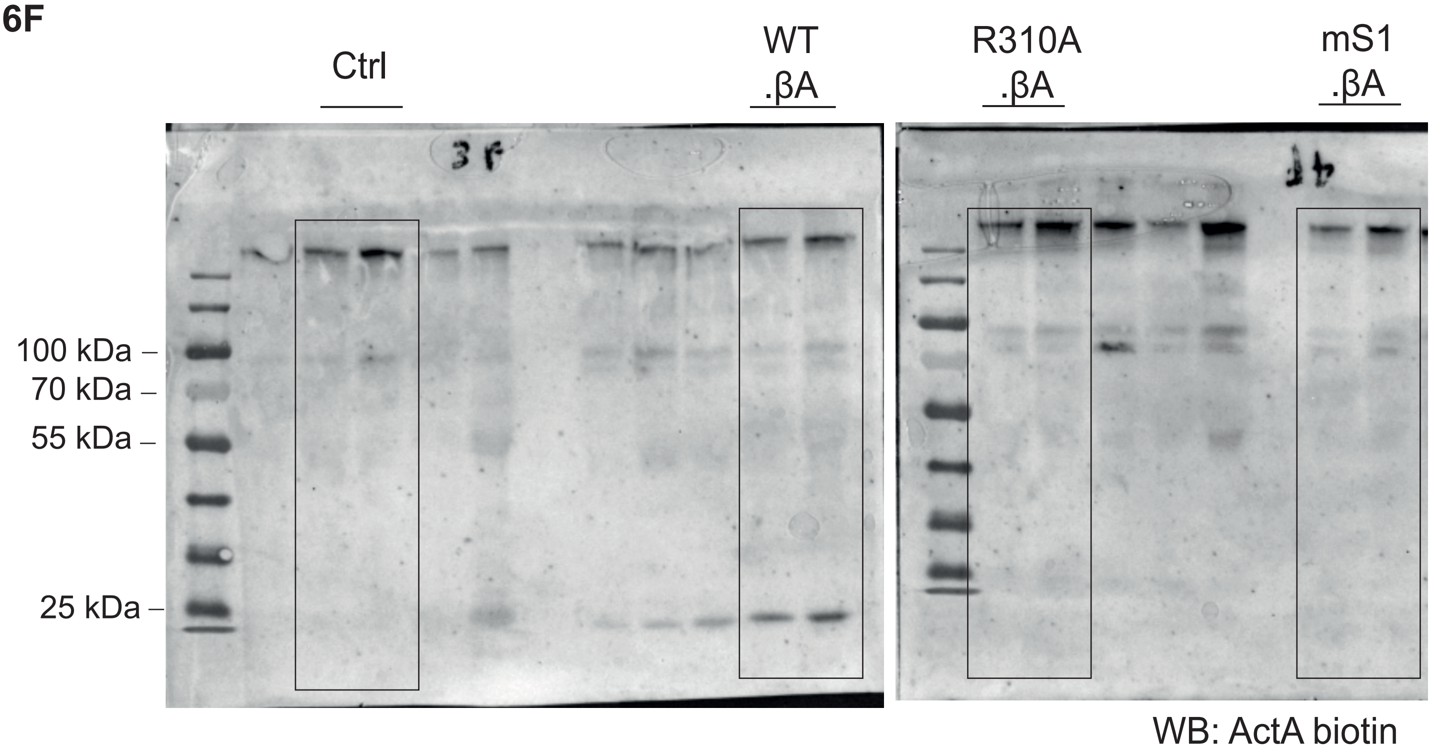
Ctrl
WT .βA
R310A .βA
mS1 .βA
100 kDa
70 kDa
55 kDa
25 kDa
WB: ActA biotin

S1B

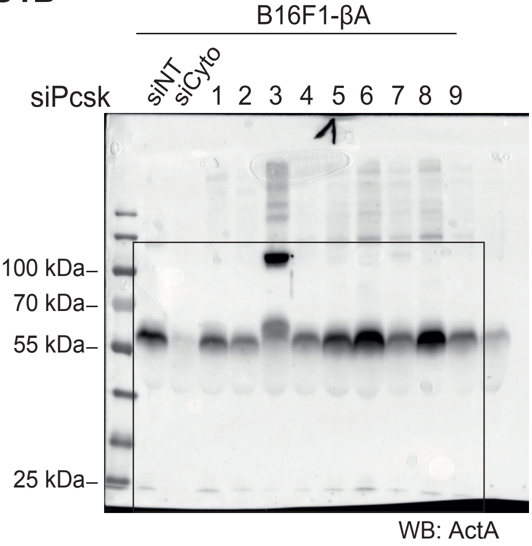

S1G

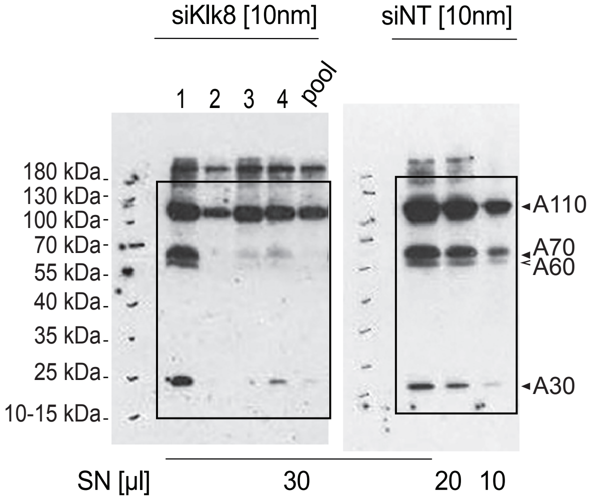

S1D

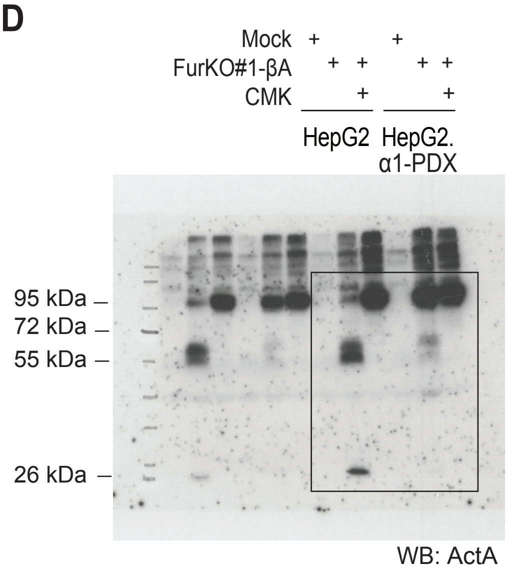

S1I

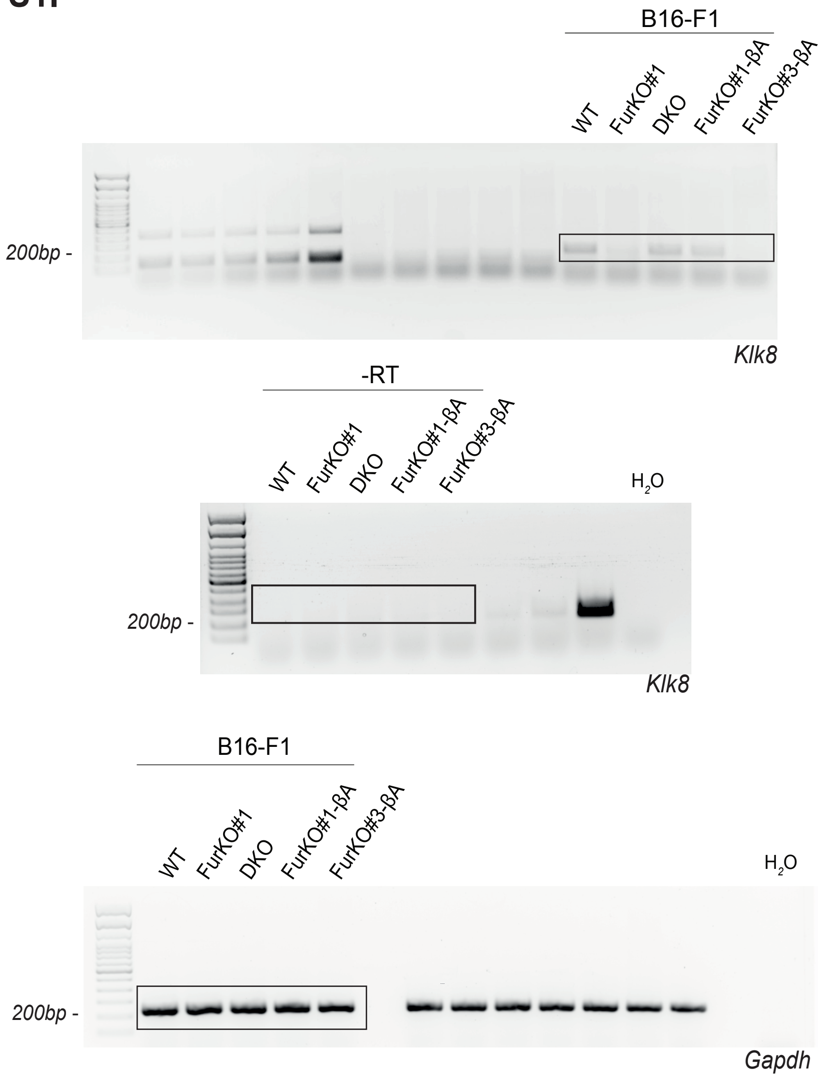

S1F

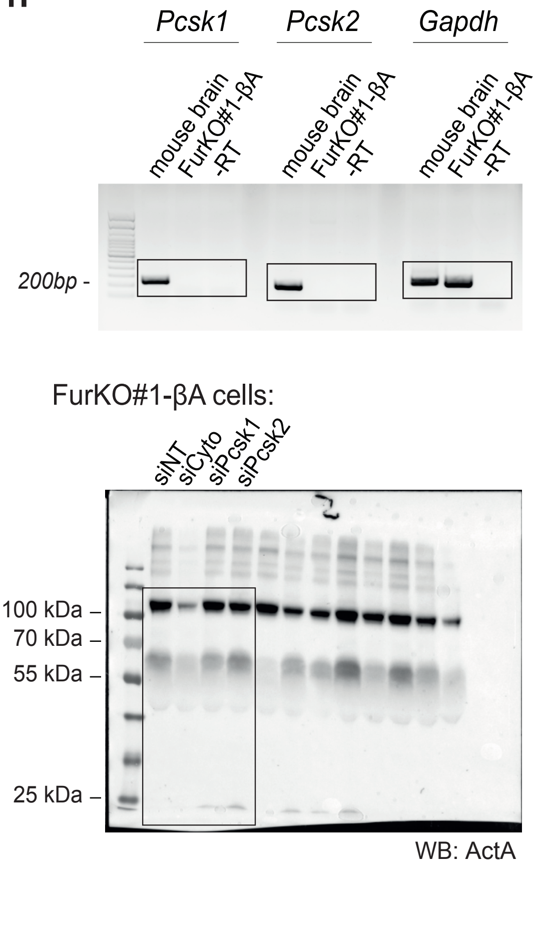

S2A

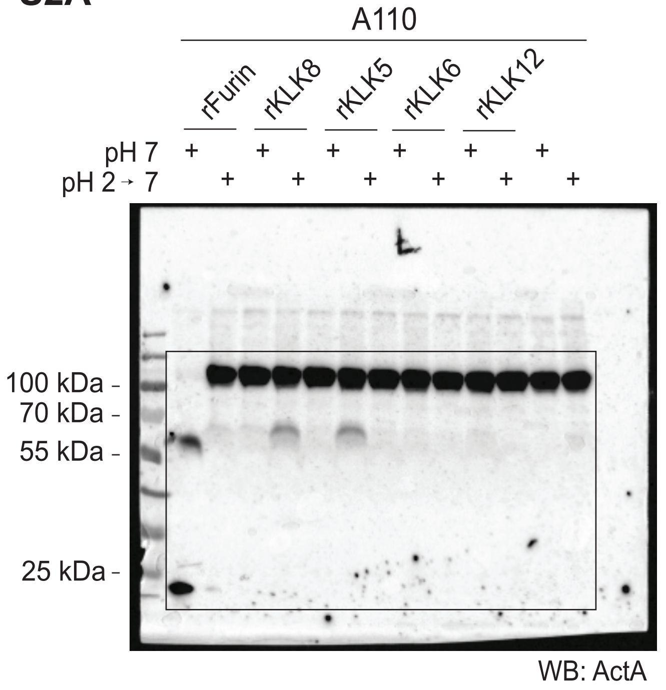

S2B

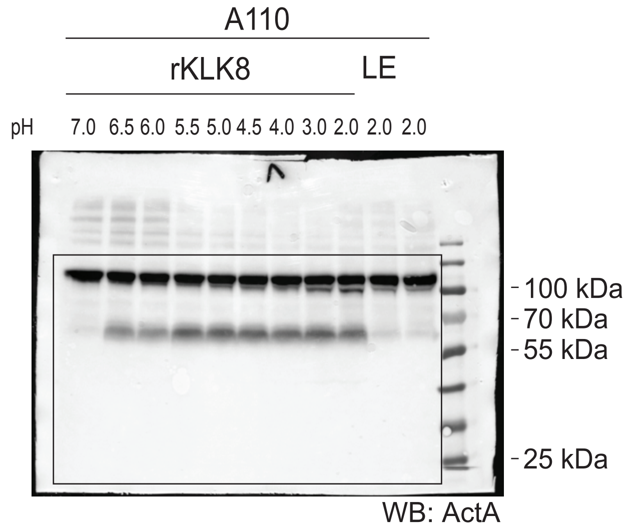

S2G

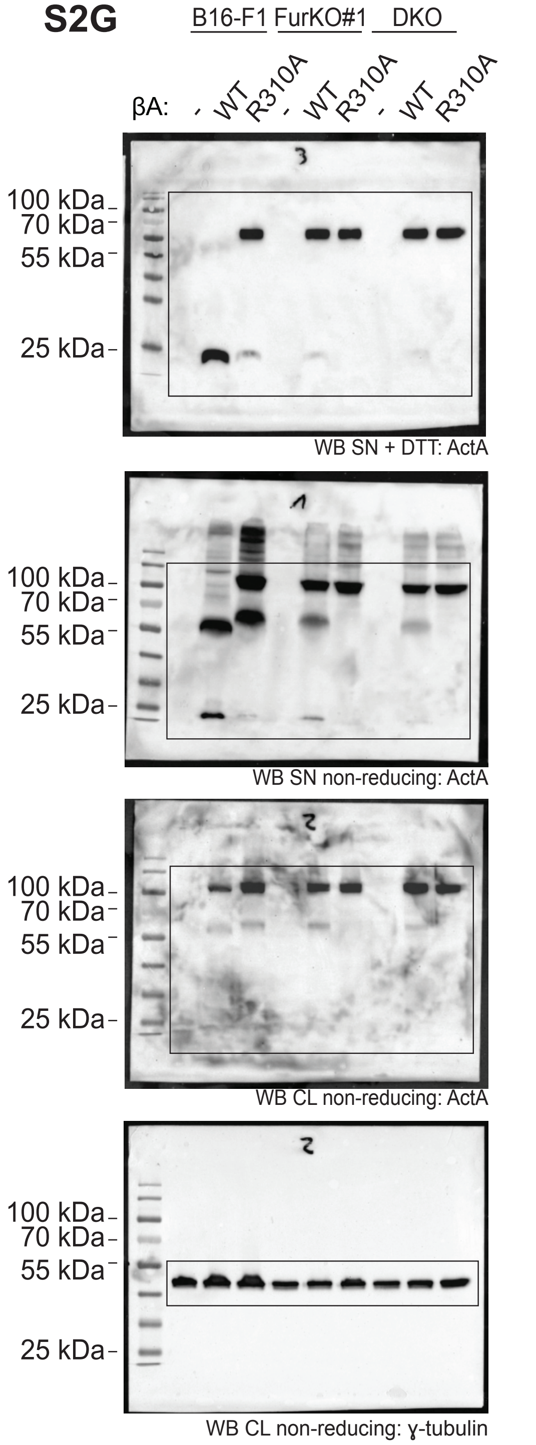

S2D

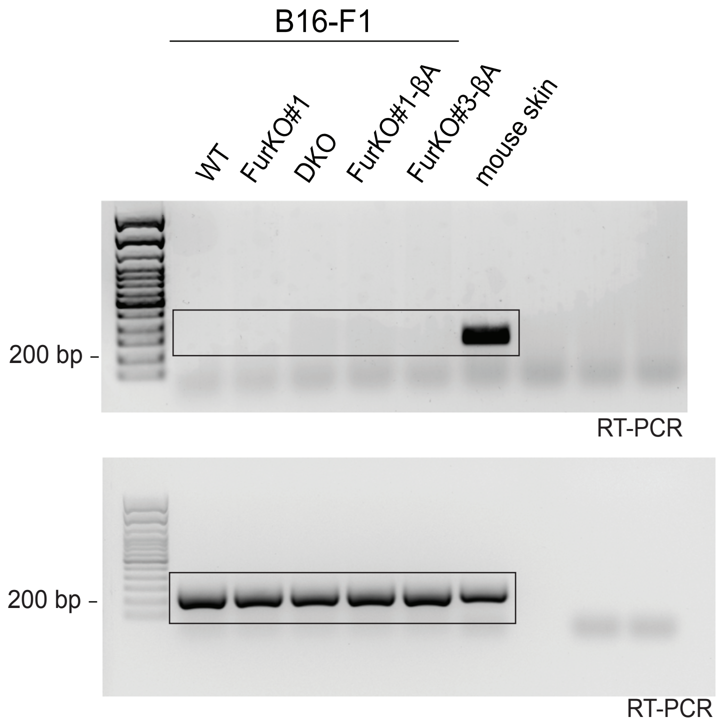

Supplement: Supplementary file 7 — Source Data [file 41467_2025_57661_MOESM7_ESM.zip › Source Data - uncropped blots.pdf]
